# Recent advances in local anesthetic drug delivery systems based on natural polymers

**DOI:** 10.3389/fbioe.2025.1727964

**Published:** 2026-01-07

**Authors:** Bing Guo, Qunhua Zhou, Xiangchao Zhang, Qiuyang Ma, Ming Ma, Tao Wang

**Affiliations:** 1 Department of Anesthesiology, Shenyang Chest Hospital, Shenyang, China; 2 Department of Medical Chemistry, Medical School, Zhengzhou Health College, Zhengzhou, China; 3 Comprehensive Department of Tumor Radiotherapy, Shenyang Sixth People’s Hospital, Shenyang, China

**Keywords:** local anesthetics, drug delivery system, natural polymers, hyaluronic acid, chitosan, alginate

## Abstract

The core objective of pain management is to effectively alleviate pain while ensuring safety, sustainability, and personalization. Local anesthetics demonstrate significant advantages in analgesia, yet their clinical application is constrained by short half-lives and the risk of cardiotoxicity and neurotoxicity when administered in high doses. The development of long-acting local anesthetics with sustained-release formulations has become a research focus. Traditional carriers like liposomes often suffer from rapid initial release and drug diffusion, leading to limited analgesic duration and low drug loading capacity. Natural polymers, however, offer distinct advantages as drug delivery systems. They exhibit excellent biocompatibility and biodegradability, breaking down into harmless byproducts in the body to prevent tissue accumulation and immune reactions. With natural origins and cost-effectiveness, polymers such as chitosan and sodium alginate also demonstrate mucosal adhesion properties to prolong drug retention at injection sites. Their highly modifiable molecular structures allow chemical adaptation to different local anesthetics. Recent advancements in microfluidics and 3D printing have optimized drug loading and controlled-release performance in natural polymer composite systems, showcasing significant clinical translation potential. This review summarizes all studies on natural polymer-based local anesthetic delivery systems that include experimental validation, animal experiments, and clinical trials, sourced from PubMed and the ClinicalTrials.gov database over the past 5 years and outlines future application prospects, providing innovative approaches for long-acting analgesia.

## 1 Introduction

Local anesthetic drugs (LADs) are a group of agents applied locally to the human body to block the generation and conduction of local nerve impulses, thereby causing temporary loss of sensory functions (e.g., pain, touch, and temperature sensation) in local tissues such as the skin, mucous membranes, and nerve trunks. They are administered via routes including transdermal, rectal, subcutaneous, intranasal, transmucosal, and sublingual administration, which can achieve effective local drug concentrations with minimal systemic exposure. They have no significant impact on consciousness or systemic physiological functions, thus greatly reducing the risk of systemic adverse reactions (Barkin, 2013; Derry et al., 2017; Gudin and Nalamachu, 2020; Sawynok, 2014). Local anesthetics exhibit temporary and reversible effects: after drug withdrawal, the function of local nerves gradually recovers to normal without permanent nerve damage. Owing to these advantages, local anesthetics are widely used not only in the treatment of acute pain (e.g., in gynecological, obstetric, and dental surgeries) but also in the management of chronic pain. The mechanism of local anesthetics involves binding to Na^+^ channels on the nerve membrane, reversibly and temporarily blocking nerve impulse conduction to produce analgesic effects and alleviate pain. However, traditional local anesthetics and conventional administration methods have several limitations. With a half-life of only 2–3 h, a single injection of local anesthetics fails to achieve long-term analgesia in clinical practice (Wei et al., 2020; Zhao et al., 2019). To meet the demand for prolonged analgesia, clinical approaches such as repeated injections, patient-controlled analgesia (PCA) pumps, or *in vivo* catheter implantation are adopted. Nevertheless, long-term catheter retention easily causes infections, and repeated administration increases patient discomfort, limiting their clinical application. Therefore, the development of long-acting local anesthetic sustained-release formulations and delivery systems has become a research focus (Ma et al., 2023).

## 2 Drug delivery systems (DDSs) for local anesthetics

Most local anesthetics (e.g., bupivacaine, lidocaine, and ropivacaine) exhibit significant clinical efficacy but have relatively short durations of action and can induce dose-dependent neurotoxicity and cardiovascular symptoms (Long et al., 2022). To achieve slow drug release, prolong analgesic duration, and reduce neurotoxicity, researchers worldwide have conducted extensive work on developing sustained-release DDSs for local anesthetics (de Paula et al., 2010; Wang et al., 2019). The goal is to reduce the dose of anesthetics while enhancing their absorption and permeability, maintaining effective drug concentrations at the target site for an extended period, and minimizing clearance. Based on the requirements for multiple anesthetic effects and different types of pain management, DDSs are generally classified into three categories: rapid-efficacy, long-efficacy, and high-efficacy (Li et al., 2021; Rodrigues da Silva et al., 2020). They can also be divided into microscale (microspheres and microneedles) and nanoscale delivery systems according to size. Research on microspheres for local anesthetic delivery dates back to around 1981. Wakiyama et al. (Wakiyama et al., 1981; 1982) encapsulated tetracaine and dibucaine in polymer systems and found that microspheres with smaller sizes could effectively regulate drug release *in vitro* and *in vivo* (Dai et al., 2018; Wakiyama et al., 1981). Microneedles are microscale needle arrays; due to their minimally invasive and self-administrable properties, they serve as effective carriers in DDSs, particularly for transdermal delivery of local anesthetics. Studies have systematically explored their feasibility as an alternative to traditional local anesthetic delivery methods (Baek et al., 2017; Castilla-Casadiego et al., 2021; Maurya et al., 2019; Priya and Singhvi, 2022). Microneedles differ from most drug delivery carriers in terms of morphology and corresponding effects. The combination of various nanocarriers with existing microneedle delivery systems is expected to significantly expand the applications of microneedles (Hao et al., 2017; Khan et al., 2022). Nanoscale DDSs, with their smaller size advantage, are more compatible with the nanostructured biological environment, facilitating cell penetration, improving bioavailability, and prolonging retention time. They play a crucial role in local anesthetic delivery (He et al., 2020; Moradkhani et al., 2018; Peptu et al., 2015; Zorzetto et al., 2016). However, nanoscale DDSs still have limitations, such as the burst release effect caused by their high surface area-to-volume ratio. These issues may require further solutions, including material modification to enhance carrier stability, process improvement to increase drug encapsulation uniformity, and composite/modification to construct sustained-release barriers. Additionally, multi-dimensional collaborative optimization should be achieved by customizing solutions based on administration routes.

## 3 Carrier materials for local anesthetic drug delivery systems

Carriers Materials for local anesthetic DDSs can be classified into liposomes, inorganic nanoparticles, and polymers based on their raw material composition (Table 1).

**TABLE 1:** Comparison of advantages and disadvantages of carriers for local anesthetic drug delivery.

Parameter	Lipid-based carriers (e.g., Liposomes, nanoemulsions)	Inorganic nanoparticles (e.g., Gold NPs, mesoporous silica NPs)	Synthetic polymers (e.g., PLGA, PCL)	Natural polymers (e.g., Chitosan, alginate)
Biocompatibility	Excellent–Phospholipid components are similar to cell membranes, resulting in excellent biocompatibility	Moderate–Inert materials have low intrinsic irritancy but may require surface modification (e.g., PEGylation) to reduce immunogenicity; unmodified particles may cause inflammation	Moderate to High–Generally good biocompatibility, but acidic degradation products of some polymers (e.g., PLGA) may cause chronic inflammation	Excellent–Derived from nature (e.g., chitin, seaweed), they exhibit excellent compatibility with human tissues and low local irritation
Biodegradability	Good–Lipid components can be metabolized by endogenous enzymes (e.g., phospholipases), with no long-term retention risk	Poor–Most are chemically stable and difficult to biodegrade, posing potential risks of long-term accumulation (except for a few like hydroxyapatite)	Moderate to High– Can be designed to be biodegradable​ (e.g., PLGA degrades into lactic and glycolic acid); degradation rate can be precisely tuned	Excellent– Can be completely degraded by human enzyme systems or hydrolysis; metabolites are non-toxic, with no risk of tissue accumulation
Drug loading efficiency/Capacity	Moderate to High–High encapsulation efficiency for lipophilic drugs (e.g., bupivacaine); loading capacity for hydrophilic drugs is relatively weaker	Excellent–Extremely high surface area (especially mesoporous materials), enabling high drug loading via adsorption/encapsulation; suitable for various drugs	Excellent–High drug loading capacity can be achieved through particle design (nanoparticles, microspheres); drugs can be loaded via physical encapsulation or chemical conjugation	Moderate to High –Depends on polymer-drug interactions; can be significantly improved via chemical modification or optimized preparation methods
Controlled release performance/Duration	Moderate to Excellent–Can achieve sustained release from hours to days (e.g., Exparel® up to 72 h); but often exhibits initial burst release. Release kinetics are highly influenced by lipid composition and structure	Excellent–Precise and prolonged release can be achieved by controlling size, porosity, and surface chemistry; release is steady with low risk of burst release	Excellent–Enables sustained and steady release over days to weeks; release profile is highly controllable by tuning polymer parameters (e.g., molecular weight, crystallinity)	Moderate–Provides sustained release for hours to a day; release kinetics often follow first-order patterns, offering less precise control compared to synthetic polymers
Stability	Moderate (Poor)– A major weakness. Prone to oxidation, hydrolysis, and particle aggregation; often requires stringent storage conditions (e.g., cold chain, protection from light)	Excellent– Exceptional physicochemical stability; resistant to temperature, pH changes, and light; long shelf life, low batch-to-batch variation	Excellent–Chemically stable, long shelf life, good batch-to-batch consistency	Moderate to Poor–Sensitive to temperature, enzymes, and pH; relatively poor stability, potential for drug leakage or polymer degradation
Preparation and Cost	Moderate– Production processes (e.g., thin-film hydration, extrusion) are established but complex; controlling particle size uniformity at scale is challenging, leading to higher costs	Moderate– Raw materials are readily available, preparation methods (e.g., sol-gel) are mature; but complex surface modification and purification steps can significantly increase costs	Moderate to High– Polymerization and formulation processes (e.g., emulsion-solvent evaporation) are complex, requiring specialized equipment and expertise, resulting in higher costs	Moderate (Low)– Raw materials are abundant and low-cost; preparation methods are relatively simple; but batch-to-batch purity variability​ in natural sources poses quality control challenges
Suitable applications	Medium to long-term postoperative analgesia (e.g., infiltration anesthesia, nerve blocks)	Interventional anesthesia, multifunctional platforms (e.g., combined drug delivery and imaging), basic mechanistic research	Ultra-long-term postoperative analgesia​ (weeks), scenarios requiring precise dosing	Mucosal delivery, analgesic wound dressings, anesthesia around sensitive tissues
Key advantages	Excellent biocompatibility, availability of marketed products (successful clinical translation)	High stability, high drug loading, precise release control	Superior control over release kinetics and longest duration	Top-tier biocompatibility and safety, often inherent bioactivity (e.g., chitosan’s antibacterial property)

### 3.1 Liposomes

Nanoliposome-based local anesthetic DDSs are the most mature and were the first to be clinically applied. Exparel™ (Pacira Pharmaceuticals, Parsippany-Troy Hills, New Jersey, USA) (Chelly et al., 2024; Coppens et al., 2019) is the first bupivacaine liposome sustained-release formulation approved by the U.S. Food and Drug Administration (FDA) for postoperative local analgesia, bunionectomy, hemorrhoidectomy, interscalene nerve block, and adductor canal and sciatic nerve blocks (Hussain et al., 2021). However, the analgesic efficacy of Exparel™ is controversial, which may depend on factors such as surgical type, pain intensity, and expected onset time of analgesia. The latest liposomal formulation for acute pain management is a combination of bupivacaine and meloxicam (Zynrelef™, Heron Therapeutics, San Diego, California, USA) [60]. Initially approved by the FDA in 2021, it is used during midline incision closure in abdominal surgery, open hernia repair, total knee arthroplasty, and bunionectomy. This approval was based on positive results from phase III clinical studies in these pain models. Compared with conventional bupivacaine, Zynrelef™ significantly reduces postoperative pain and opioid consumption within 72 h postoperatively (Blair, 2021; Viscusi et al., 2019a; Viscusi et al., 2019b). In 2024, the indications of Zynrelef™ were expanded to include open shoulder and spinal surgeries. However, Zynrelef™ has only been on the market for a short time, and its future clinical use requires further evaluation. Furthermore, all liposomal formulations face the issue of skeletal stability: lipids degrade into harmful metabolites during long-term storage. Excessive lysophospholipids and other lipid fragments can bind to red blood cells, leading to fatal hemolysis. To overcome the formulation limitations of existing formulation, lidocaine liposome gel formulations are under development, and animal data suggest that gel formulations may have greater application potential in humans (Leon et al., 2024). In addition, coating with chitosan or alginate may resolve this issue by stabilizing liposomes and extending their shelf life to 2 years (Abu Lila and Ishida, 2017; Kapoor et al., 2017).

### 3.2 Inorganic nanoparticles

Since inorganic nanoparticles were introduced as drug carriers, their application in local anesthetic delivery has been extensively studied, including metallic nanoparticles (Jahangiri et al., 2013; Lueshen et al., 2014; Mocanu et al., 2012; Mocanu et al., 2013; Wang et al., 2020) and non-metallic nanoparticles (Gao et al., 2019; Liu et al., 2018). Owing to their important properties such as controllable particle size distribution, high affinity for pathological cells, and controllable delivery characteristics, inorganic nanoparticles are considered an indispensable strategy for targeted delivery of local anesthetics (Amaldoss et al., 2022; Li et al., 2019). Among metallic nanoparticles, gold and silver nanoparticles are regarded as superior to other types (Mocanu et al., 2012; Mocanu et al., 2013).

### 3.3 Biodegradable polymers

As suitable materials for local anesthetic delivery, polymers are focused on due to their properties complementary to those of liposomes. Unlike liposomes, which rely on van der Waals forces and hydrogen bonds, polymers are formed via covalent bonds. This structure not only endows them with better storage stability but also maintains stability when co-administered with free local anesthetics. A large number of biocompatible and biodegradable polymers have been used to prepare sustained-release materials, including natural and synthetic polymers.

With the rapid development of manufacturing technology, sustained-release systems based on synthetic polymers have advanced significantly. The most commonly used synthetic polymers are polyesters, including poly-lactic acid (PLA), poly-ε-caprolactone (PCL), poly (lactic-co-glycolic acid) (PLGA), and peptide-drug conjugates (Duceac and Coseri, 2022; El-Say and El-Sawy, 2017; Maadani and Salahinejad, 2022; Silva Favacho et al., 2020). Their metabolites are usually non-toxic small molecules such as carbon dioxide and water, which can be safely circulated or excreted (Swider et al., 2018). However, as non-physiological exogenous substances, synthetic polymers have the drawback of inducing adverse foreign body reactions (Zorzetto et al., 2016). In contrast, natural polymers exhibit significant advantages, attracting the attention of numerous researchers.

## 4 Advantages of natural polymers as carriers in local anesthetic drug delivery systems

In local anesthetic DDSs, natural polymers exhibit irreplaceable advantages due to their natural origin, structural diversity, and high compatibility with the biological environment. They have become one of the core materials for addressing the limitations (e.g., low drug solubility, poor bioavailability, and strong toxic side effects) of traditional administration methods.

### 4.1 Excellent biocompatibility

Biocompatibility is a core requirement for drug delivery systems: materials should not induce immune rejection, inflammatory reactions, or cytotoxicity *in vivo*, ensuring that drugs act precisely on the target without damaging normal tissues. Natural polymers are mostly derived from plants/animals (e.g., cellulose, chitosan) or microorganisms (e.g., hyaluronic acid, alginate). Their chemical structures (e.g., hydroxyl and amino groups in polysaccharides, peptide bonds in proteins) are highly similar to endogenous macromolecules in the human body (e.g., glycogen, collagen), enabling them to be “recognized” by the body as “endogenous substances,” thereby significantly reducing immunogenicity and toxicity.

### 4.2 Controllable biodegradability to avoid “material accumulation” risks

Traditional synthetic polymers (e.g., polyethylene, polyvinyl chloride) are difficult to degrade *in vivo*. Organ damage may be caused by their long-term accumulation. In contrast, most natural polymers can be degraded into small-molecule fragments under physiological conditions (e.g., enzymes, acid-base environments) and eventually excreted via metabolic pathways without the need for secondary surgical removal. More importantly, their degradation rate can be “on-demand adjusted” by regulating material molecular weight, cross-linking degree, or environmental conditions (e.g., pH, enzyme concentration). For example, chitosan-alginate composite microspheres used for long-acting sustained release can extend the drug release cycle from several days to several weeks by adjusting the cross-linking degree, while the material degrades slowly to avoid accumulation.

### 4.3 Abundant functional groups for “precise drug loading and targeted delivery”

Natural polymers typically have a large number of active functional groups on their molecular chains (e.g., hydroxyl [-OH], amino [-NH_2_], carboxyl [-COOH], aldehyde [-CHO]). These groups provide convenient chemical sites for “drug loading” and “targeted modification,” enabling optimization of DDS performance through multiple approaches such as efficient drug loading (to prevent premature leakage) and targeted modification. For instance, when chitosan nanoparticles are loaded with bupivacaine, the chitosan molecular chains are rich in amino (-NH_2_) and hydroxyl (-OH) groups. Bupivacaine contains a tertiary amine group (protonated to carry a positive charge under physiological pH) and a hydrophobic aromatic ring. Electrophilic interactions dominate the construction of the delivery system (nanoparticles), while hydrophobic interactions and hydrogen bonds synergistically achieve efficient drug encapsulation and initial stabilization. During the release phase, the body fluid environment first weakens the electrostatic forces, causing system swelling; subsequently, water molecules disrupt hydrogen bonds, leading to drug dissociation; finally, the drug is released through diffusion, with hydrophobic interactions serving as the final barrier to ensure sustained release. This cascade effect of multiple interactions collectively enables the continuous and controlled release of bupivacaine, effectively prolonging the anesthesia duration (Deng et al., 2022).

### 4.4 Favorable physicochemical properties for multiple administration scenarios

By adjusting preparation processes (e.g., emulsification-crosslinking, electrospinning, self-assembly), natural polymers can form drug-loaded carriers of various morphologies (e.g., microspheres, nanoparticles, hydrogels, fiber membranes). The stability, swelling property, and drug release behavior of these carriers can be flexibly adjusted to meet the needs of multiple administration scenarios, including oral, injection, and local administration (e.g., skin, mucous membranes).

### 4.5 Wide availability, low cost, and environmental friendliness

Compared with synthetic polymers (e.g., polylactic acid, polyethylene glycol), natural polymers are derived from abundant and renewable raw materials such as agricultural waste (e.g., corn starch, straw cellulose), aquatic by-products (e.g., chitosan extracted from shrimp and crab shells), or microbial fermentation products (e.g., hyaluronic acid). Their production costs are significantly lower, facilitating industrial-scale production. Meanwhile, the production process of natural polymers (e.g., extraction, purification) consumes less energy, and the materials are biodegradable in the natural environment without causing “white pollution,” aligning with the trend of green medicine development.

### 4.6 Natural bioactivity enabling “synergistic therapy” potential

Some natural polymers possess inherent bioactivities (e.g., anti-inflammatory, antibacterial, and tissue repair-promoting effects). When combined with drugs, they can achieve a synergistic effect of “carrier function + drug efficacy,” enhancing therapeutic outcomes. For instance, chitosan has natural antibacterial properties, its drug-loaded microspheres can inhibit bacterial proliferation while delivering local anesthetic, strengthening anti-infective effects (Hanif et al., 2021; Harrison et al., 2021). Hyaluronic acid can promote the proliferation of skin fibroblasts; its drug-loaded hydrogels can also simultaneously improve skin barrier function while delivering drugs (Chen et al., 2024; Jeong et al., 2025).

The advantages of natural polymers in DDSs essentially stem from the combination of their “biological compatibility” and “functional flexibility.” Although some natural polymers (e.g., chitosan) have limitations such as poor water solubility and uncontrollable degradation rates, their performance can be further optimized through chemical modification (e.g., grafting with polyethylene glycol) or composite with other materials (e.g., blending with synthetic polymers) to broad the application prospects in the field of local anesthesia (Table 1).

## 5 Research exploration of natural polymers as carriers in local anesthetic drug delivery systems

In the research of local anesthetic drug delivery systems, the application of natural polymers as carriers has garnered increasing attention. The following sections of this article will delve into different types of natural polymers, such as hyaluronic acid, cellulose, chitosan, alginates, and gelatin, exploring their practical applications in local anesthetic drug delivery (Table 2).

**TABLE 2:** Recent Studies of Natural polymer-based drug delivery systems for local anesthetics.

Polymers	Carriers	Drugs	Preparations	Release duration	Animal/Clinical trial info	Main effect	Ref.
Hyaluronic acid (HA)	Lipid emulsion/HA	Bupivacaine	Hydrogel	∼80% release by 20 h (BLE), ∼100% by 68 h (HA-BLE)	Rat sciatic nerve block model	A 5-times greater anesthetic area under the curve and an anesthetic duration that was twice as long as controls	Davis et al. (2020)
	HA	Lidocaine	Dissolving microneedles (DMNs)	Onset within 10 min	Pig carcass insertion test	Fast onset time and minimally invasive administration methods	Yang et al. (2020)
	HA/hydroxypropyl chitin thermo-sensitive hydrogel (HPCH)	Ropivacaine	Hydrogel	Thermal sensation block: 17.7 ± 0.7 h (R-HPCH-HA) vs. 5.7 ± 0.8 h (R HCl). Motor block: 6.8 ± 0.8 h vs. 3.5 ± 0.8 h	Rat sciatic nerve block model	Prolonged the local analgesic effect in rats without notable side effects	Qiao et al. (2022)
	Solid-lipid	Bupivacaine	Microparticles/Hydrogel	Designed for 72 h release	-	Hyaluronic acid hydrogel is required for lowering injection forces as well as minimizing clogging events	Wojtalewicz et al. (2022)
	HA	Lidocaine	Dissolving microneedles (DMNs)	DMNs dissolved completely after 3 min	Rabbit model	Li-DMNs can obtain a local anesthesia effect at a relatively lower dose, and there was no oral mucosal irritation in rabbits	Lee et al. (2023)
	Poloxamer (PL) 407/PL 338/HA	Bupivacaineand ropivacaine	-	-	-	Hyaluronic acid can promote increased structural stabilization by hydrophilic interactions between hyaluronic and micellar corona	Sepulveda et al. (2023)
	Carboxymethylcellulose (CMC-ADH)/oxidized hyaluronic acid (OHA)	Ropivacaine	Heterogeneous hydrogel microspheres	*In vitro*: >160 h. *In vivo* sensory block: 48 h; motor block: 36 h	Rat sciatic nerve block model	Long-term retention and drug release *in vivo*	Li et al. (2024)
	Poly (vinyl pyrrolidone) (PVP)/poly (vinyl alcohol) (PVA)/sodium hyaluronate (SH)	Ropivacaine	Dissolving microneedles (DMNs)	-	-	Both formulations were also able to deliver more than 60% of the RPL contained in the DMNs into the epidermis, dermis, and receiver compartment	Ramadon et al. (2024)
	Poly (vinylpyrrolidone) (PVP)/hyaluronic acid (HA)	Lidocaine	Dissolving cambered microneedle (MNs) patch	Sustained effect ≥30 min	Porcine eyeball, heel skin of mice	Exhibited effective analgesic effects for local anesthesia on both heel skin and eyeball, with the sustained anesthetic effect lasting for at least 30 min	Liu et al. (2025)
	Core-shell microgels/HA	Lidocaine	Hydrogel	Sustained release for 24 h	Golden hamster model	Prolonged wet adhesion, antibacterial action, analgesia, and tissue regeneration	Wu et al. (2025b)
	Poloxamer 407 (P407)/oxidized hyaluronic acid (OHA)	Bupivacaine	Hydrogel	*In vitro*: up to 8 days. *In vivo* mechanical pain threshold: 32 h; thermal pain threshold: 48 h	Rat sciatic nerve block model	Provided sustained bupivacaine release for up to 8 days *in vitro* andextended the mechanical pain threshold for 32 h and thermal pain threshold for 48 h *in vivo*	Yang et al. (2025)
Cellulose	Hydroxypropyl methylcellulose	Lidocaine	Buccal films	-	-	The mucoadhesive and mechanical properties were effectively modified	Eleftheriadis et al. (2020)
	Carboxymethylcellulose (CMC)/ball-milled glutinous starch (BMGS)	Lidocaine	Films	Release rate: 2.05% ± 0.21%–7.55% ± 1.08%/min. Penetration rate: 3.48 ± 0.28–8.04 ± 0.64 μg/cm^2^/min^1^/^2^	-	Provide sustained delivery of water-soluble active ingredients	Limpongsa et al. (2021)
	Core polymer (PVP-K30)/ethyl cellulose (N7)	Lidocaine	Microneedles	Up to 9 h	Porcine skin model	PCP microneedles of lidocaine hydrochloride could constantly release the drug for up to 9 h in the skin tissue	Jakka et al. (2022)
	Ethyl cellulose, 2% Carbopol, and 5% eudragit	Lidocaine	Buccal patch	-	Human double-blind randomized controlled trial (split-mouth study)	More efficient in controlling the injection pain than articaine	Panahandeh et al. (2024)
	Sodium carboxymethyl cellulose (NaCMC)/cross-linked gelatin	Lidocaine	Microneedles (MNs)/microcapsules (MCs)	Initial burst within 10 min, sustained release over 240 min	-	Displayed significant antimicrobial properties and good biocompatibility, with cell viability exceeding 86%	Bahmani et al. (2025)
	Sodium carboxymethylcellulose	Ropivacaine	Gel	-	Human retrospective analysis (75 patients)	Effectively prolong the duration of pain relief	Deng et al. (2025)
Chitosan	Hydroxypropylmethylcellulose (HPMC)/sodium alginate (SA)	Lidocaine	Mucoadhesive dosage	-	-	Optimized formulation TL5 exhibited dosage stability up to 6 months at 75% relative humidity and retained drug contents	Hanif et al. (2021)
​	Hexanoic acid-treated electrospun chitosan membranes (HA-ESCM)	Bupivacaine	Biofilms	72 h	-	Membranes release both therapeutics for 72 h, and release profile can be tailored by loading concentration	Harrison et al. (2021)
​	Chitosan	Ibuprofen	Microspheres	-	Human clinical trial (60 patients)	Comparatively better analgesic and anti-inflammatory properties with drastic reduction of pain, swelling, trismus, and also Had a reliable wound healing property	Karthik and Balamurugan (2021)
​	Glycosylated chitosan (GCS)/mesoporous silica nanoparticles	Ropivacaine	Nano-scaffold	50% release in 2 h, 90% in 12 h (with ultrasound)	Rat sciatic Nerve Chronic Constriction Injury (CCI) Model	Using ultrasound Stimulation to trigger drug release, enhance controllability, and improve drug delivery efficiency	Qi et al. (2021)
​	Chitosan/PLGA/Pluronic™ F-127	Benzocaine	Nanoparticles/hydrogels	-	Cell viability >70% (fibroblasts, keratinocytes) - *In vitro* only	Both the nanoparticles and hydrogels were able to modulate BZC delivery and increase drug permeation	Campos et al. (2022)
​	Chitosan/genipin/poly (ε-caprolactone) (PC)	Bupivacaine	Nanocapsules	*In vitro*: 99.2% ± 1.12% release in 36 h. *In vivo* pain response: improved for at least 7 days	Mouse model	CS-GP hydrogel and CS-GP/PC polymeric hydrogel improved the skin permeation of BPV 3-fold and 5-fold in 24 h, respectively	Deng et al. (2022)
​	Chitosan (CS)/β-sodium glycerophosphate (β-GP)	Ropivacaine	Liposomal gel (Lip/Gel) composite	Retained *in vivo* for 10 days	Rat model	NIR-responsive and effectively solve the shortcomings of traditional local injections, reduce the toxicity and side effects of free Rop	Hou et al. (2022)
​	Chitosan/porous gelatin/calcium alginate	Lidocaine	Microspheres (CPAMs)	Rapid release induced by swelling	-	Acted as fast-release dressings for faster pain control and better coagulation properties	Ma et al. (2022)
​	Chitosan/poly (MMA-co-HEMA-cl-EGDMA) (CsPMH)	Bupivacaine	Nanogels	Carrier 7: ∼91% release in 24 h. Carrier 6: ∼62% release in 12 h	-	Provide the controlled delivery of anesthetic drugs	Nagella et al. (2023)
​	Polycaprolactone (PCL)/chitosan (CS)	Procaine	Composite scaffolds	Release examined up to 1 week	-	CS contributes to the sustained release dynamic of Procaine	Dedeloudi et al. (2024)
Alginate	Alginate	Lidocaine	Nanoemulsions	90% release through PDMS and porcine skin in 24 h, able to release over 48 h	-	Extended-release profile is highly favorable in transdermal drug delivery	Sarheed et al. (2021)
​	Alginate/Supramolecular phenolic-based nanofillers (SPFs)	Lidocaine	Hydrogel	Sustained release for 14 days *in vivo*	*In vivo* (Rat)	Demonstrated sustained release for 14 days *in vivo*, validating the potential for long-term anesthesia	Pan et al. (2023)
​	Sodium alginate (SA)/Glycerol/polyvinyl alcohol (PVA)	Lidocaine	Dissolving microneedle	95% release within 24 h	Golden hamster model	Accelerated mouth healing, improves drug delivery and patient comfort	Elhabal et al. (2025)
​	Alginate microparticles/gellan gum/collagen matrix	Bupivacaine	Hydrogel	Simultaneous release with different kinetics	-	The microparticles played a crucial role in maintaining the hydrogel’s integrity in the presence of both drugs and enabled their controlled and simultaneous release	Garrido et al. (2025)
​	Celecoxib@Laponite-dopamine-alginate-Pluronic F-127@ropivacaine (CLDAFR)	Ropivacaine	Hydrogel	Controlled drug release *in vitro*	Rat model of chronic pain and MI/R injury	Treating chronic pain-exacerbated MI/R injury by precisely targeting the SCG and providing a sustained anti-inflammatory and analgesic effect	Wu et al. (2025a)
​	Alginate/Gelatin/riboflavin	Bupivacaine	Hydrogel	Sustained release exceeding 72 h *in vitro*	Sheep cadaver model	Provided a moderate burst decrease and sustained release that exceeded 72 h and decreased drug-induced cytotoxicity	Steverink et al. (2022)
​	Sulfobutyl ether β-cyclodextrin (SCD)/Hyaluronic acid (HA)	Lidocaine	Hydrogel	Significantly prolonged analgesic effect (∼2 h longer than conventional BVC in orofacial pain models)	Orofacial pain models in mice (formalin, carrageenan, postoperative pain)	Exhibits significantly prolonged analgesic effect and low cytotoxicity	Zhou et al. (2022)
​	L-Arginine and 2-Hydroxypropyl-β-Cyclodextrin (2HPβCD)	Lidocaine	Deep eutectic solvents	Cumulative transdermal amount reached 252.4 μg/cm^2^ (45% water) at 8 h	-	With good drug solubility and permeation enhancing effects	Lv et al. (2023)
Gelatin	Gelatin-methacryloyl (GelMA)	Lidocaine	Hydrogel microneedles (MNs)	Prolonged anesthetic effect	Rat spared nerve injury (SNI) model	Enhance and prolong the anesthetic effect of LiH without dermatosis-related side effects	Zhao et al. (2022)
	Gelatin/NHS-PEG-NHS	Bupivacaine	Hydrogel	Sustainable release profile	Mouse sciatic nerve block model	The analgesic effect was greatly improved *in vivo*, and there was no obvious evidence of permanent inflammation or nerve damage in the block site’s sections	Zhang et al. (2024)

### 5.1 Hyaluronic acid (HA)

Hyaluronic acid (HA) is a naturally occurring negatively charged glycosaminoglycan with multiple properties, including biocompatibility, biodegradability, water absorption, water retention, and high viscosity. It is naturally degraded *in vivo* via enzymatic mechanisms (Chen et al., 2024). HA is mainly present in the dermis of the skin, synovial fluid, heart valves, vitreous humor of the eye, cartilage, and extracellular matrix. Hyaluronic acid has emerged as a promising material for local anesthetic drug delivery systems. Its biocompatibility, biodegradability, and versatility allow for the development of various formulations, including hydrogels, microneedles, and nanofibers, tailored to specific applications. Recent research highlights the potential of HA-based systems to improve drug efficacy, prolong drug release, reduce side effects, and provide targeted delivery in diverse fields like wound healing, pain management, and treatment of skin conditions (Harrer et al., 2021).

#### 5.1.1 Hyaluronic hydrogels as local anesthetic carriers

**FIGURE 1:**
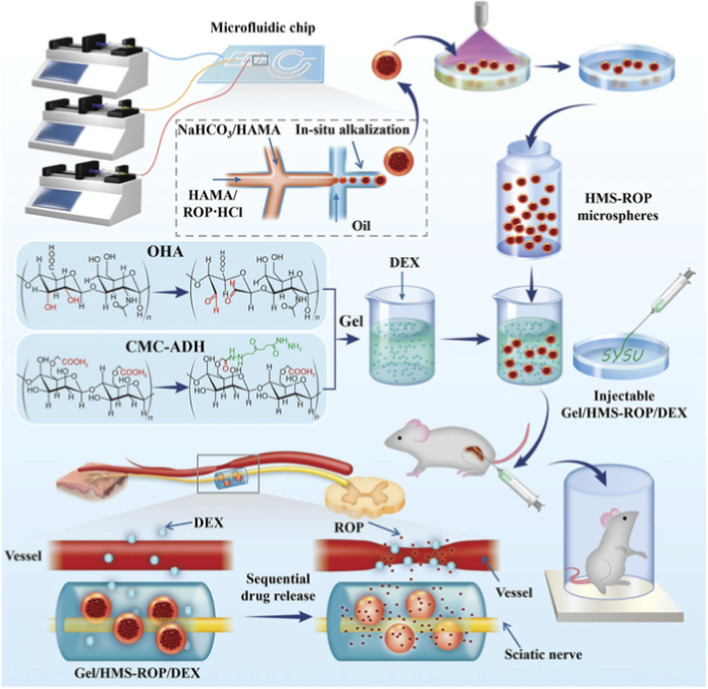
Schematic illustration of preparing a Gel/HMS-ROP/DEX delivery system for prolonging the duration of regional nerve blockade. Reproduced with permission from ref (Li et al., 2024); CC BY 4.0. Copyright © 2024 by the authors.

Davis B et al. entrapped bupivacaine-loaded lipid emulsion (BLE) droplets (generated via high-speed homogenization) into a cross-linked HA hydrogel system to form an injectable composite gel formulation (HA-BLE). The results showed that the incorporation of HA hydrogel protected BLE droplets from the *in vivo* environment, significantly enhancing anesthetic efficacy (Davis et al., 2020). Qiao et al. combined hydroxypropyl chitin thermosensitive hydrogel (HPCH) with HA to construct a ropivacaine sustained-release system. Utilizing the negative charge property of HA, which interacts with positively charged ropivacaine, the system improved administration efficiency. In a rat sciatic nerve block model, its duration and efficacy were significantly superior to those of ropivacaine hydrochloride, with low cytotoxicity (Qiao et al., 2022). Sepulveda et al. studied the morphology and structure of polymeric formulations based on poloxamer and its binary mixture with hyaluronic acid as drug delivery systems for local anesthetics (bupivacaine and ropivacaine). The addition of HA promoted increased structural stabilization through hydrophilic interactions, affecting drug release (Sepulveda et al., 2023). Yang et al. developed a dual-crosslinked hydrogel (Bup/PO) composed of amidated poloxamer 407 (P407) and oxidized hyaluronic acid (OHA) via oxime bonds and hydrophobic bonds. This dual-crosslinking mechanism improved hydrogel stability, mechanical properties, and drug release. A rat sciatic nerve block model showed that Bup/PO hydrogel significantly prolonged the mechanical pain threshold for 32 h and the thermal pain threshold for 48 h (Yang et al., 2025). Wu et al. developed a cross-linked HA hydrogel (PHB) embedded with core-shell microgels, a combined drug hydrogel system. The sustained release of epidermal growth factor (EGF) and lidocaine from PHB hydrogel lasted for 24 h, consistent with the time window required for oral mucosal repair. It synergistically prolonged wet adhesion, antibacterial effects, analgesia, and tissue regeneration, providing a clinical solution for oral mucosal repair (Wu et al., 2025b). Another local anesthetic delivery system, Gel/HMS-ROP/dexmedetomidine (DEX), consists of an injectable self-healing hydrogel matrix (GEL) prepared from modified carboxymethyl cellulose (CMC-ADH) and oxidized hyaluronic acid (OHA), combined with heterogeneous hydrogel microspheres (HMS-ROP). It exhibits long-term retention and *in vivo* drug release capabilities: *in vitro* drug release lasted for over 160 h, and *in vivo* sensory and motor block durations were 48 and 36 h, respectively, enabling prolonged local anesthesia (Li et al., 2024) (as shown in Figure 1).

#### 5.1.2 Hyaluronic microneedles as local anesthetic carriers

**FIGURE 2:**
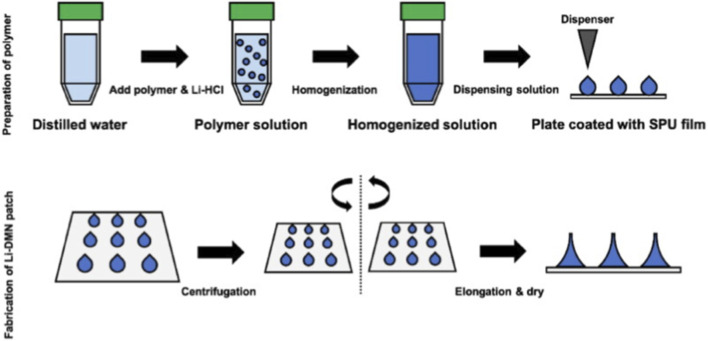
Flow chart of HA-loaded lidocaine for the preparation of dissolving MNs. Reproduced with permission from ref (Yang et al., 2020) CC BY 4.0. Copyright © 2020 by the authors.

Yang et al. prepared Lidocaine-Loaded Dissolving Microneedle (Li-DMNs) using HA as the backbone, enabling minimally invasive transdermal delivery of lidocaine. With a mechanical strength of 0.116 N per needle, it can effectively balance the penetration efficiency and drug release rate. *In vivo* experiments showed that Li-DMNs had a rapid onset of action (within 10 min) and a long duration of effect without causing various skin adverse reactions, demonstrating high clinical safety (Yang et al., 2020) (as shown in Figure 2). Hu et al. fabricated conductive microneedles (MNs) using polyaniline and HA and combined them with iontophoresis (ITP) for the delivery of dextran macromolecules. Conductive MNs assisted by ITP improved the penetration of dextran in the skin, fat, muscle, and cartilage (Hu et al., 2022). A novel and safe oral Li-DMN has been confirmed to have potential applications in large animal and clinical trials. It was prepared by centrifugal lithography using a lidocaine hydrochloride-HA mixed solution. The oral lidocaine hydrochloride-encapsulated DMNs completely dissolved within 3 min after oral administration, achieving local anesthetic effects at a relatively low dose, which can meet the requirement of dental surgeries (Ramadon et al., 2024). Dissolving microneedles loaded with ropivacaine hydrochloride (DMN-RPL) are composite drug delivery platforms prepared using centrifugation or air pressure chamber methods with several polymers (e.g., polyvinylpyrrolidone [PVP], polyvinyl alcohol [PVA], and sodium hyaluronate [SH]). The research demonstrate the feasibility of delivering RPL as a local anesthetic via DMNs and intradermal routes, aiming to minimize pain and discomfort during administration and improve patient experience (Ramadon et al., 2024). A rapidly dissolving curved microneedle patch (MNs@Lido) composed of PVP and HA was used as a delivery system for lidocaine (Lido) in local anesthesia. MNs@Lido had sufficient rigidity to penetrate the cornea without causing subsequent damage, and the created corneal needle hole channels fully healed within 24 h. MNs@Lido exhibited effective analgesic effects for local anesthesia of heel skin and eyeballs, with a duration of anesthetic effect of at least 30 min, opening up new possibilities for the treatment of ophthalmic diseases (Liu et al., 2025). Ramadon et al. compared HA and poly (vinyl pyrrolidone) as backbone polymers for lidocaine-loaded dissolving microneedles. Although both polymers showed promise, HA demonstrated a safe concentration of the drug permeating into the systemic circulation, making it a viable option for intradermal lidocaine delivery.

Hyaluronic acid has become an interesting and important polymer due to its beneficial properties such as solubility, biocompatibility, and biodegradability. As research continues, HA is poised to play an increasingly significant role in advancing local anesthesia and targeted drug delivery strategies. However, several issues need to be further addressed, such as reducing the injection force of HA hydrogels and minimizing clogging events. Future research directions will focus on improving the properties of HA as a pharmaceutical excipient, exploring various possibilities for its chemical modification, and designing and conducting *in vivo* and clinical studies (Ramadon et al., 2024; Wojtalewicz et al., 2022).

### 5.2 Cellulose

Cellulose is one of the most widely distributed and abundant natural polymers in nature, present in plants, animals, and bacteria. It possesses properties such as renewability, biocompatibility, biodegradability, and non-toxicity (Karimian et al., 2019). These properties make it suitable for drug delivery applications, particularly for local anesthetics. Recent advancements in cellulose-based drug delivery systems have shown promising results in improving the pharmacological properties of local anesthetics. Cellulose derivatives, such as hydroxypropyl methylcellulose (HPMC) and hydroxyethyl cellulose (HEC), have gained attention due to their biocompatibility, biodegradability, and ability to form hydrogels. Among these, HPMC is most widely used in delivery systems. In early studies, Hoare et al. used a rheological polymer blend of HPMC and HA as a carrier for bupivacaine in rat sciatic nerve block models. The duration of sensory nerve block was extended by approximately 3-fold. This blend exhibited no cytotoxicity and only induced mild short-term inflammatory reactions at the injection site (Hoare et al., 2010). A CMC-BMGS matrix prepared by blending carboxymethyl cellulose (CMC) with ball-milled glutinous starch (BMGS) showed favorable matrix quality, thickness, moisture content, hygroscopicity, and mechanical and mucoadhesive properties. It exhibited advantages in modulating the sustained release and penetration of the water-soluble active ingredient lidocaine hydrochloride from the mixed matrix (Limpongsa et al., 2021). Deng et al. used sodium carboxymethyl cellulose as a sustained-release agent in the formulation of ropivacaine gel, which effectively prolonged the duration of pain relief and reduced postoperative discomfort at the donor site after costal cartilage harvesting for ear reconstruction (Deng et al., 2025).

Cellulose is more commonly fabricated into microneedles for drug delivery. Some researchers prepared lidocaine-loaded fish scale nanocellulose composite microneedles (MNs) with the mechanical strength can reach 0.23 N/needle. These MNs could successfully penetrate the stratum corneum and absorb moisture to separate the drug-polymer matrix, thereby releasing the drug from the polymeric material. This method not only replaces traditional transdermal administration but also increases skin permeability, improving the bioavailability of local anesthetics (Bian et al., 2025; Medhi et al., 2017). Jakka developed Polymer Coated Polymeric (PCP) microneedles to explore the feasibility of gradual release of active ingredients from the system. Animal tissue models were used to investigate the potential applications of these PCP microneedles in skin and intravitreal administration. PCP microneedles combined with lidocaine hydrochloride could sustain drug release in skin tissue for up to 9 h (Jakka et al., 2022). Bahmani S developed a dual-release transdermal delivery system using a cross-linked hydrogel composed of gelatin and sodium carboxymethyl cellulose (NaCMC) as the matrix, with lidocaine hydrochloride as the model drug. The system was designed with an immediate drug release mechanism via microneedles (MNs) and a sustained release mechanism via microcapsules (MCs). The manufacturing process involved casting the hydrogel into an MN mold and embedding MCs in the backing layer. The MNs had a conical shape and strong mechanical properties, exhibiting excellent penetration ability. *In vitro* release profiles showed an initial burst release within 10 min, followed by sustained release over 240 min. Additionally, the system exhibited significant antibacterial properties and good biocompatibility, with a cell viability of over 86%, confirming its potential as a safe and effective transdermal delivery platform (Bahmani et al., 2025).

**FIGURE 3:**
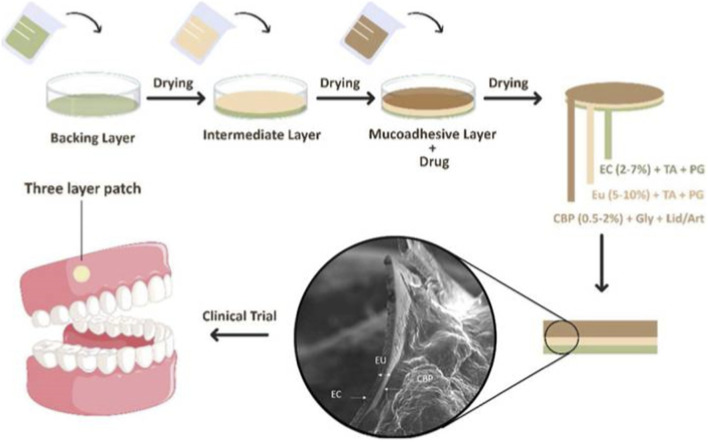
Schematic demonstration of the preparation process and clinical trial. Reproduced with permission from ref (Panahandeh et al., 2024) CC BY-NC-ND 4.0. Copyright © 2024 by the authors.

Cellulose can also be fabricated into mucoadhesive films. Georgios et al. prepared HPMC-based films and developed an oral mucosal drug delivery system capable of co-delivering local anesthetics and non-steroidal anti-inflammatory drugs (NSAIDs) to the oral mucosa for the treatment of oral diseases (Eleftheriadis et al., 2020). Panahandeh et al. optimized the formulation of a 3-layer buccal patch, which consisted of 4.72% ethyl cellulose, 2% Carbopol, and 5% Eudragit. Clinical evaluation results showed that loading lidocaine in the optimized formulation was more effective in controlling injection pain than articaine (Panahandeh et al., 2024) (as shown in Figure 3).

The versatility of cellulose derivatives, combined with innovative delivery methods such as nanoparticles, hydrogels, and microneedles, offers the potential for improved therapeutic outcomes. Future research should focus on optimizing these systems for specific clinical applications, ensuring safety and efficacy in local anesthetic delivery.

### 5.3 Chitosan

Chitosan is the second most abundant natural cationic polysaccharide after cellulose. Derived from the deacetylation of chitin, it exhibits properties such as non-toxicity, biodegradability, biocompatibility, antibacterial activity, hemostatic effect, and anticancer activity, making it suitable for various drug delivery systems and widely used in drug delivery (Ali and Ahmed, 2018; Yu et al., 2024). To date, chitosan has been studied as a carrier for local anesthetics via multiple administration routes, including oral, transdermal, injection, and transmucosal routes (Deng et al., 2022; Hou et al., 2022; Karthik and Balamurugan, 2021). Owing to its diverse functional roles, chitosan can act as a coating component, carrier matrix, surface modifier, or penetration enhancer in DDSs and is often used in combination with other polymers.

**FIGURE 4:**
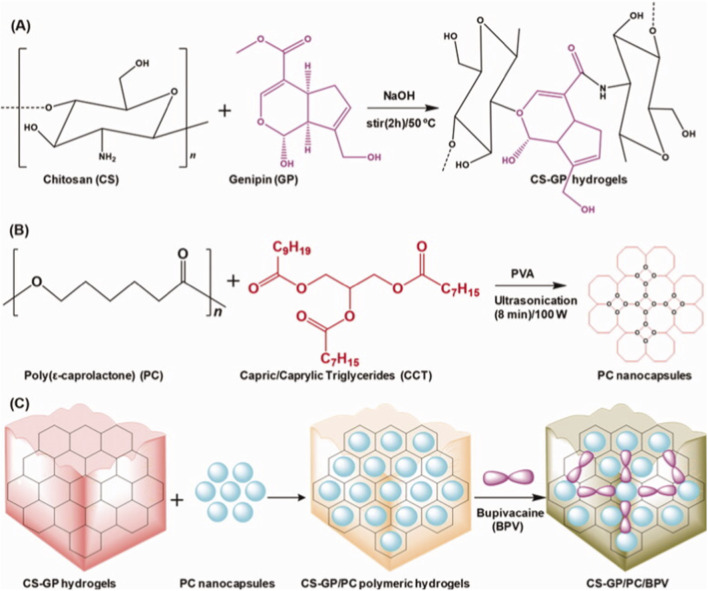
Schematic representation of **(A)** the preparation of chitosan (CS) and genipin (GP) crosslinked CS-GP hydrogels, **(B)** the preparation of PC nanocapsules, and **(C)** CS-GP hydrogels loaded PC nanocapsules to bupivacaine (BPV). Reproduced with permission from ref (Deng et al., 2022) CC BY 4.0. Copyright © 2022 by the authors.

Zhang et al. prepared chitosan (CH)-coated polycaprolactone (PCL) nanoparticles for the co-delivery of ropivacaine and dexamethasone complexes. *In vitro* and *in vivo* experiments after transdermal administration in mice showed that chitosan-PCL nanoparticles could serve as effective drug carriers, prolonging and enhancing the anesthetic effect of ropivacaine (Zhang et al., 2017). Campos EVR coated the surface of thermosensitive Poly (lactic-co-glycolic acid) (PLGA) nanoparticles with chitosan for sustained-release local delivery of benzocaine (BZC). Physicochemical and cytological experiments confirmed improved drug permeability and cell viability. This hybrid system shows promise as a delivery system for long-term topical anesthetic therapy (Campos et al., 2022). Qi et al. designed a novel nanoscaffold GCS-MONPs@RV based on glycosylated chitosan (GCS)-encapsulated mesoporous silica nanoparticles (GCS-MONPs) for the controlled release of ropivacaine (RV). This system utilized ultrasound stimulation to trigger drug release, significantly improving drug delivery efficiency compared with traditional methods (Qi et al., 2021). In another experiment, Ma RR et al. prepared a lidocaine-containing dressing using chitosan, porous gelatin, and calcium alginate. This dressing rapidly released 90.5% of lidocaine within 18 min, followed by slow release of the remaining lidocaine, achieving better pain control (Ma et al., 2022). Deng et al. developed a skin drug delivery system by combining nanocapsules (CS-GP/PC) based on chitosan and genistein hydrogels with the local anesthetic bupivacaine (BPV), which prolonged anesthesia and pain relief (Deng et al., 2022) (as shown in Figure 4). Hou et al. developed a near-infrared (NIR)-responsive composite delivery system for ropivacaine (Rop). The *in situ* gel component composed of chitosan and β-glycerophosphate sodium (β-GP) effectively reduced the release of responsive Rop liposomes (Rop@Lip), addressing the limitations of traditional local injection and reducing the toxicity and side effects of free Rop. It serves as an excellent photoresponsive delivery system for analgesic drugs (Hou et al., 2022). Another chitosan-based nanogel drug carrier, chitosan/poly (MMA-co-HEMA-cl-EGDMA) (CsPMH), reported by Nagella SR, was also shown to exhibit better sustained-release effects (Nagella et al., 2023).

Chitosan itself possesses antibacterial bioactivity, enabling it to exert antibacterial effects while delivering anesthetics, expanding its application scope. Harrison et al. prepared chitosan biofilms loaded with cis-2-decenoic acid (C2DA) and bupivacaine, which relieved pain while preventing infections (Harrison et al., 2021). Hanif et al. prepared a chitosan-based mucoadhesive for the delivery of butoconazole iodide and lidocaine hydrochloride. Utilizing the mucoadhesive and antibacterial properties of chitosan, this adhesive can be used for the treatment of oral diseases such as pharyngitis (Hanif et al., 2021). Recently, Dedeloudi et al. prepared procaine (PRC)-loaded polycaprolactone (PCL) and PCL-chitosan composite scaffolds via bioprinting, investigating the physicochemical stability and mechanical integrity of the scaffolds. *In vitro* drug release studies showed that chitosan contributed to the sustained-release of PRC (Dedeloudi et al., 2024).

While chitosan-based local anesthetic drug delivery systems show great promise, there remain many challenges to be addressed. The need for precise control over drug release rates, stability of formulations, and scalability of production are critical factors that must be addressed. Future research should focus on optimizing the physicochemical properties of chitosan formulations and exploring novel combinations with other materials to enhance therapeutic outcomes.

### 5.4 Alginates

Alginates are natural polysaccharide materials derived from brown algae, with widely available raw materials. As a promising candidate for drug delivery, it has emerged due to its biocompatibility, biodegradability, and ability to form hydrogels, thus attracting extensive research. Its unique ion-sensitive gelation property simplifies the drug loading process. It also exhibits controllable sustained drug release performance, high drug-loading capacity, and universality. Although it has good overall biocompatibility, high-purity alginate (purity >99%) is costly. Industrial-grade products may retain small amounts of impurities such as brown algae proteins and polysaccharides, which may induce mild local inflammatory reactions when used for injection or implantation.

An innovative drug-loaded formulation of alginate-based nanoemulsions for sustained release of lidocaine reported by Sarheed et al. The lidocaine-loaded polymeric nanoemulsion formulation could release the drug *in vivo* for 48 h, exhibiting long-lasting anesthetic effects with significantly reduced toxicity compared with traditional administration methods. This sustained-release property shows advantages in transdermal delivery of the local anesthetic lidocaine (Sarheed et al., 2021).

**FIGURE 5:**
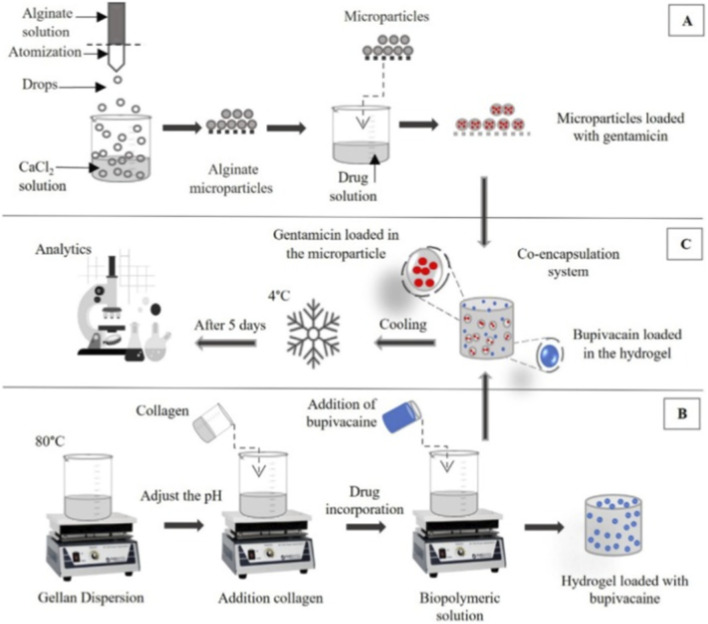
Schematic representation of the methodology used to prepare the dual-drug delivery systems. Reproduced with permission from ref (Garrido et al., 2025) CC BY 4.0. Copyright © 2025 by the authors. **(A)** Preparation of alginate microspheres loaded with gentamicin, **(B)** preparation of carrageenan/collagen hydrogels loaded with bupivacaine, and **(C)** preparation of a dual-drug delivery system.

Hydrogels can endow various therapeutic agents with sustained-release properties, and the ion-sensitive gelation property of alginates makes them important sustained-release carriers. Pan et al. loaded the anesthetic lidocaine into SPF-integrated alginate hydrogels, which exhibited sustained release for 14 days *in vivo*, verifying its potential for long-term anesthesia (Pan et al., 2023). Wu et al. designed an injectable Pluronic/alginate composite hydrogel loaded with celecoxib and ropivacaine (celecoxib@Laponite-dopamine-alginate-Pluronic F-127@ropivacaine, CLDAFR). The CLDAFR hydrogel exhibited excellent biocompatibility, thermosensitive gelation properties, and controlled drug release *in vitro*. *In vivo*, it could accurately target the superior cervical ganglion (SCG) and provide sustained anti-inflammatory and analgesic effects, which can be used for the treatment of myocardial ischemia-reperfusion (MI/R) injury complicated with chronic pain (Wu et al., 2025a). Elhabal et al. designed microneedle (MN) patches (SA/PVA) composed of sodium alginate (SA), glycerol, and polyvinyl alcohol (PVA) loaded with lidocaine for the management of oral ulcerative mucositis (OUM). *In vitro* studies showed enhanced permeability of SA/PVA and significantly increased lidocaine release (reaching 95% within 24 h). *In vivo* evaluation demonstrated significant pain relief, reduced inflammation (decreased levels of TNF-α and NF-κB), enhanced expression of the anti-inflammatory cytokine IL-10, and regulated angiogenesis via downregulation of VEGF, thereby accelerating oral healing and complete epithelial repair. This innovative system goes beyond traditional anesthetic administration, providing a painless and targeted therapeutic platform for improved outcomes (Elhabal et al., 2025). Exploration of advanced biomaterials for dual drug delivery is also in progress. Garrido et al. incorporated alginate microparticles containing the antibiotic gentamicin into a collagen hydrogel matrix and directly loaded the local anesthetic bupivacaine. This design combines antibacterial and analgesic effects in a single platform, enabling simultaneous release of the two drugs from an advanced dual-delivery system based on a hydrogel-microparticle composite. It highlights the potential of this hydrogel-microparticle composite as an advanced material for wound dressings, which can promote healing while providing local pain relief (Garrido et al., 2025) (as shown in Figure 5).

Despite the promising results from alginate-based local anesthetic delivery systems, several challenges remain. The optimization of drug loading, release kinetics, and stability of the formulations is crucial for their successful application. Additionally, the potential for inflammatory responses at the injection site must be carefully evaluated to ensure patient safety.

### 5.5 Cyclodextrins

Cyclodextrins (CDs) are non-toxic cyclic oligosaccharides composed of D-glucopyranose units linked by α-1,4-glycosidic bonds. They are obtained by the degradation of amylose via cyclodextrin glucosyltransferase. CDs have a hydrophilic outer shell and a hydrophobic inner cavity, which can encapsulate hydrophobic drugs to enhance their water solubility and prevent degradation. They also can improve drug absorption, enhance bioavailability, mask drug odors, control drug release, reduce gastrointestinal irritation, and decrease drug toxicity (Zhang and Ma, 2013). The most common CDs include α-CD, β-CD, and γ-CD, each differing in the number of glucose units and cavity size. β-CD and its derivatives, such as 2-hydroxypropyl-β-cyclodextrin (HP-β-CD), are frequently used in pharmaceutical formulations due to their suitable cavity size for many drug molecules and improved water solubility (Hammoud et al., 2019; Omtvedt et al., 2021).

In early studies, researchers prepared levobupivacaine complexes containing maltosyl-β-cyclodextrin (G2-β-CD) using CD-based delivery systems. The results showed that the complex of levobupivacaine and G2-β-CD significantly prolonged the anesthetic effect in rat intrathecal and sciatic nerve block models (Karashima et al., 2007). Additionally, studies reported that in rabbits, the duration of epidural anesthesia with bupivacaine combined with HP-β-CD was nearly twice as long as that with bupivacaine alone (Freville et al., 1996). Similar results were observed in sciatic nerve block models. In sheep, bupivacaine-CD epidural anesthesia was also confirmed to be more effective than bupivacaine alone (Estebe et al., 2002). Zhou et al. prepared sulfobutyl ether β-cyclodextrin (SCD)/hyaluronic acid (HA) hydrogels loaded with lidocaine (LDC). Compared with free lidocaine, SCD/HA-LDC showed significantly prolonged analgesic effects and lower cytotoxicity (Zhou et al., 2022). Recently, researchers prepared deep eutectic solvents (DESs) with various water contents (40%–60%) using L-arginine as the hydrogen bond acceptor (HBA) and 2-hydroxypropyl-β-cyclodextrin (2HPβCD) as the hydrogen bond donor (HBD) at a mass ratio of 4:1. The physicochemical properties of these DESs in transdermal drug delivery systems (TDDSs) were investigated, confirming that these non-irritating, viscous liquid-like DESs have good drug solubility and penetration-enhancing effects, showing broad application prospects in the fields of drug dissolution and transdermal delivery systems (Lv et al., 2023). Steverink et al. developed robust and cytocompatible gelatin hydrogels prepared via riboflavin-mediated photocrosslinking. Oxidized β-cyclodextrin was incorporated into the hydrogel formulation to form dynamic bonds with tyramine functional groups, thereby achieving self-healing behavior and shear resistance. The implantability of the hydrogel was confirmed in cadaveric instrumented spinal surgery. Furthermore, hydrogels were loaded with bupivacaine crystals to provide sustained release beyond 72 h *in vitro*. The use of crystallized bupivacaine decreased cytotoxicity compared with bupivacaine HCl. The present formulation can aid in enabling opioid-free analgesia following instrumented spinal surgery (Steverink et al., 2022). de Freitas Domingues et al. prepared bupivacaine hydrochloride (BVC)-sulfobutyl ether-β-cyclodextrin (SBEβCD) complexes (BVC-SBEβCD). Compared with conventional BVC, BVC-SBEβCD showed prolonged analgesic effects (de Paula et al., 2010).

The drug-loading advantages of CDs stem from their unique cavity structure and good biocompatibility, which can significantly improve the solubility and dissolution rate of hydrophobic drugs, enhance drug stability, reduce degradation and oxidation, and minimize toxic side effects.

### 5.6 Gelatin

Gelatin is a naturally water-soluble polymer extracted via hydrolysis of animal collagen (e.g., bovine hide, pigskin, and tendon). Owing to its good biocompatibility, biodegradability, and unique gelation/film-forming properties, it is widely used in various drug delivery systems (e.g., microspheres, hydrogels, capsule shells, and wound dressings) for oral, injection, and local administration. It has outstanding advantages include high biocompatibility and low immunogenicity, controllable biodegradability with safe degradation products, excellent gelation and moldability (enabling diverse dosage forms), strong drug-loading capacity and universality, and good mucoidhesion prolonging drug retention time.

Zhao et al. designed a high-dose hydrogel microneedle (MN) system made of gelatin-methacryloyl (GelMA) for sustained delivery of lidocaine hydrochloride (LiH). The use of a backing layer reservoir significantly improved the drug-loading capacity of GelMA MNs. *In vivo* biosafety evaluation in rats showed that LiH/GelMA MNs could enhance and prolong the anesthetic effect of LiH, with no skin disease-related side effects or behavioral disorders observed during the experiment. This confirms that GelMA MNs for transdermal delivery of LiH are an effective, safe, and simple treatment method, providing a better option for long-term local analgesia (Zhao et al., 2022).

Zhang et al. prepared an injectable hydrogel via crosslinking gelatin with NHS-PEG-NHS for regional nerve block. The hydrogel had a porous three-dimensional network structure, high drug-loading capacity, and a sustainable drug release profile, achieving long-term analgesia (Zhang et al., 2024). In addition, researchers have used advanced technical approaches to combine gelatin with polymers such as cellulose and cyclodextrins to construct novel composite drug-loading platforms, adapting to the anesthetic needs of different clinical scenarios (Bahmani et al., 2025; Steverink et al., 2022).

### 5.7 Advantages and disadvantages of different natural polymeric materials as local anesthetic carrier

Different natural polymers exhibit distinct advantages and limitations as carriers for local anesthetics, and their selection requires a comprehensive consideration of the needs of specific clinical applications (as shown in Table 3). Hyaluronic acid (HA) and gelatin are top choices for safety due to their excellent biocompatibility and biodegradability. However, HA’s susceptibility to rapid enzymatic degradation *in vivo* can compromise long-term drug release, while gelatin suffers from poor thermal stability and mechanical strength. Chitosan, as the only natural cationic polysaccharide, offers inherent benefits like antibacterial activity and mucoadhesion, which are advantageous for preventing infection and enhancing local retention. Nonetheless, its poor solubility at physiological pH limits its broader application. Alginate and cyclodextrin excel in drug loading and controlled release. Alginate forms mild hydrogel networks capable of high drug loading and prolonged release, whereas cyclodextrin significantly enhances the solubility of hydrophobic drugs and prolongs efficacy through molecular encapsulation. However, they face challenges related to weak gel mechanical strength (alginate) and drug selectivity (cyclodextrin), respectively. The primary drawback of cellulose is its poor biodegradability, though chemical modifications can yield various functional materials. Current optimization strategies primarily focus on chemical modifications (e.g., cross-linking, derivatization) and physical blending (creating composite materials) to overcome individual weaknesses and synergize strengths. For instance, combining chitosan with PLGA nanoparticles or blending alginate with gelatin can synergistically improve the stability and functionality of the carrier system. Future research will likely emphasize developing intelligent, responsive, and multifunctional composite carrier systems to achieve more precise, prolonged, and safe local anesthesia therapy.

**TABLE 3:** Comprehensive comparison of characteristics of various natural polymers as local anesthetic carriers.

Characteristic dimension	Hyaluronic acid (HA)	Cellulose	Chitosan	Alginate	Cyclodextrin (CD)	Gelatin
Key physicochemical properties	Anionic polysaccharide, high viscosity, strong hydrophilicity and water retention, unique ion-sensitive gelation properties	Abundant hydroxyl groups, can be derivatized (e.g., HPMC, HEC, CMC), good film-forming and gelation properties	Natural cationic polysaccharide, rich in amino groups, possesses antibacterial and hemostatic activity, strong mucoadhesion	Anionic polysaccharide, forms mild hydrogels (ion-sensitive gels) via cross-linking with cations like Ca^2+^	Cyclic oligosaccharides, hydrophobic cavity, hydrophilic shell, can encapsulate hydrophobic drugs, significantly increasing their solubility	Hydrolyzed product of animal collagen, thermosensitive gelation properties, excellent film-forming ability and biocompatibility
Common local anesthetics	Bupivacaine, Lidocaine, Ropivacaine	Lidocaine	Ropivacaine, Bupivacaine, Lidocaine	Lidocaine, Bupivacaine, Ropivacaine	Bupivacaine, Ropivacaine, Lidocaine	Lidocaine, Bupivacaine
Biocompatibility	Excellent, widely present in human extracellular matrix, non-immunogenic	Good, no corresponding degrading enzymes in humans, but the material itself is non-toxic and harmless	Good, but the acidic microenvironment from its degradation products may cause mild inflammation	Fair, but algal protein impurities in low-purity products may cause immune reactions	Excellent, safe pharmaceutical excipient	Excellent, hydrolyzed product of human collagen, low immunogenicity
Drug loading efficiency/Capacity	Medium to High, as a hydrogel matrix can effectively physically encapsulate drugs	Medium, limited by molecular structure, loading rate usually below 30%, but can be optimized via modification	Medium to High, can load drugs via multiple mechanisms like ionic cross-linking, hydrophobic interactions	High, its hydrogel network structure allows high drug loading, with good universality	High, utilizes its hydrophobic cavity to encapsulate drugs, offering a unique and efficient loading method	High, its three-dimensional network structure can effectively encapsulate drugs
Controlled release ability (Duration)	Excellent, can achieve sustained release from several hours to several days (e.g., 48 h to 14 days)	Medium, can achieve sustained release for several hours (e.g., 4–6 h)	Medium to Good, can achieve sustained release from several hours to several days (e.g., 24 h to >72 h)	Excellent, enables prolonged, steady release for days to weeks (e.g., >72 h to 14 days)	Excellent, can significantly prolong anesthesia duration (e.g., extend to 2x), with steady release	Excellent, enables long-acting sustained release (e.g., >72 h)
Biodegradability	Excellent, can be degraded by hyaluronidase *in vivo*, no residue	Poor, no corresponding hydrolytic enzymes in humans, difficult to degrade	Excellent, can be degraded by lysozyme etc., in vivo, but rate may be uneven	Excellent, undergoes hydrolytic degradation, degradation products are safe	Good, can be degraded by colonic flora	Excellent, can be degraded by proteases *in vivo*, safe without accumulation
Common formulation types	Hydrogel, Dissolving microneedles, Nanofibers	Mucosal patches, Microneedles, Films	Nanoparticles, Hydrogel, Mucoadhesive patches, Nanofiber membranes	Hydrogel, Microneedles, Microspheres, Nanoemulsions	Inclusion complexes, Hydrogels, Deep eutectic solvents	Hydrogel, Microneedles
Main disadvantages/Challenges	Rapid enzymatic degradation *in vivo* leads to premature carrier disintegration, affecting longevity; high cost of high-purity raw materials	Poor biodegradability, risk of long-term retention; strong intermolecular hydrogen bonding leads to poor solubility and processability	Poor solubility in neutral pH body fluids, prone to aggregation; degradation may produce an acidic microenvironment, causing local irritation	Weak mechanical strength of gels, prone to erosion; strong ion dependence, structure unstable in the variable ionic strength of the *in vivo* environment	Drug inclusion is selective, not universally applicable; high concentrations may cause hemolysis	Poor thermal stability, easily inactivated at high temperatures; weak mechanical properties, often requires cross-linking with other materials for reinforcement
Optimization strategies	Cross-linking modification to improve stability; development of enzyme inhibitors to delay degradation	Chemical derivatization (e.g., carboxymethylation, hydroxypropylation) to improve solubility; compounding with biodegradable polymers (e.g., gelatin)	Chemical modification (e.g., quaternization, carboxymethylation) to improve solubility; used as a coating to modify other nanoparticles (e.g., PLGA) to enhance functionality	Blending with other polymers (e.g., gelatin) to enhance mechanical properties and sustained-release characteristics; fine purification of raw materials	Structural derivatization (e.g., HP-β-CD, SBE-β-CD) to improve water solubility and safety; constructing composite systems (e.g., cyclodextrin polymers)	Chemical cross-linking (e.g., using genipin, glutaraldehyde) to improve stability; blending with other polymers (e.g., alginate) to enhance mechanical strength
Degradation-PK profile match	Excellent to Moderate Fast enzymatic degradation may limit duration for very long-term PK needs, but suitable for short-to-medium term	Poor Low biodegradability leads to potential accumulation, poorly matching desired PK profiles requiring clearance	Good Controllable enzymatic degradation rates can be tailored to match short-to-medium term PK profiles effectively	Excellent Predictable, tunable ion-mediated degradation aligns well with sustained, long-term PK profiles	Good Controlled dissociation of drug-inclusion complexes provides a release profile that matches medium-term PK.	Excellent Protease-driven degradation profile is highly compatible with sustained drug release for medium-to-long term PK.

## 6 Challenges and innovations in local anesthetic delivery systems based on natural polymers

### 6.1 Optimizing drug loading capacity and sustained release effect

Achieving optimal drug loading capacity and controlled release profiles is crucial for the success of local anesthetic delivery systems. Limited by their molecular structures, the drug loading capacity of natural polymers is much lower than that of many synthetic polymers. Additionally, the initial burst release phenomenon present in natural formulations can disrupt the intended prolonged analgesic effect and increase the risk of systemic toxicity. For example, in chitosan hydrogels at neutral pH, the dissociation of protonated amino groups may trigger the rapid release of local anesthetics like ropivacaine. Furthermore, the dynamic physiological environment, such as pH levels, enzyme activity, and temperature fluctuations, can complicate the sustained and stable release of drugs (Beach et al., 2024; Li et al., 2023). Recent advances in materials science and bioengineering provide innovative solutions to address these challenges. For instance, advanced cross-linking techniques, such as reversible covalent bonds and supramolecular interactions, have been used to enhance drug retention and prevent burst release (Hu et al., 2019). The integration of multiple dosage forms, such as combining liposomes or polymer nanoparticles with a hydrogel matrix, can achieve the dual functions of improving drug encapsulation efficiency and enabling controlled/sustained release (Valentino et al., 2024). Advanced interdisciplinary technologies (like nanotechnology, image-guided technology, and artificial intelligence (AI)-assisted design) can also be incorporated into the design of local anesthetic delivery systems (Negut and Bita, 2023). These technologies allow researchers to simulate and predict drug release kinetics under various physiological conditions, enabling the development of drug carriers tailored to specific clinical needs. Stimuli-responsive delivery systems have garnered considerable attention due to their ability to tailor drug release based on local physiological conditions. These systems utilize external triggers, including changes in pH, temperature, enzyme activity, or redox status, to achieve different release rates, making them suitable for treatment regimens requiring sequential or pulsed drug delivery (Tian and Liu, 2023).

Overcoming obstacles related to drug loading and sustained release in natural polymer-based local anesthetic delivery systems requires a multifaceted approach, such as combining cutting-edge materials science, computational tools, and smart responsive technologies. These advancements continue to pave the way for designing efficient and controllable local anesthetic delivery systems.

### 6.2 Biocompatibility and safety issues

Although the degradation products of natural polymers are generally considered non-toxic, long-term local accumulation may still induce inflammatory reactions. Variations in polymer composition, cross-linking methods, and degradation products can also pose significant safety concerns. Biocompatibility may vary significantly across different tissue types, necessitating the customization of formulations for specific clinical scenarios. One promising strategy involves embedding immunomodulators within the delivery system; this approach has shown great potential in preclinical models, where the integration of immunomodulators improved tissue compatibility and therapeutic outcomes (Liu X et al., 2024). Additionally, for enzyme-sensitive polymeric materials, degradation enzyme inhibitors can be used to fine-tune the degradation rate, synchronizing the drug release profile with therapeutic needs.

Addressing biocompatibility and safety issues requires a multidisciplinary approach integrating materials science, pharmacology, and clinical practice, prioritizing patient safety and therapeutic efficacy.

### 6.3 Mechanical stability in dynamic environments

Natural polymers often face stability challenges under physiological conditions. Insufficient structural integrity may lead to their premature degradation, limiting their effectiveness in delivering local anesthetics within dynamic environments. Thiolation modifications (e.g., thiolated hyaluronic acid) can introduce cross-linking sites to enhance the stability of HA hydrogels. Functional modifications can also be achieved through biomimetic compounding or constructing hybrid materials. For example, combining natural polymers with synthetic materials (such as PLGA, polyethylene glycol, PEG) to form “hybrid carriers” can reduce the degradation rate of the natural polymer. Furthermore, externally triggered delivery systems represent another significant advancement, allowing for precise control over mechanical stability and therapeutic delivery. For instance, laser-induced local heating can regulate drug release to achieve the desired analgesic effect. These externally triggered delivery systems combine adaptability and precision, paving the way for patient-specific pain management strategies (Thang et al., 2023). By leveraging these innovations, hydrogel-based systems are increasingly capable of overcoming the mechanical challenges posed by physiological conditions, ensuring they efficiently deliver local anesthetics even in the most demanding clinical scenarios.

### 6.4 Preparation process optimization

Innovations in preparation processes provide technical support for the development of novel drug-loaded systems. Microfluidic chips can be used to prepare core-shell microspheres with consistent particle size, effectively controlling the size distribution and improving drug loading capacity and encapsulation efficiency. Ensuring quality consistency, reproducibility, and sterility in large-scale manufacturing is crucial. Microfluidic technology is more likely to be integrated first into modern GMP continuous production lines. Advances in 3D printing have revolutionized drug delivery systems, enabling the creation of highly personalized solutions to meet individual patient needs (Liang et al., 2024). The integration of computational modeling with the 3D printing process further enhances its capabilities, reducing the traditional trial-and-error in delivery system development and accelerating clinical translation efficiency. For example, computationally optimized site-specific hydrogels have shown excellent efficacy in preclinical models, providing sustained analgesia while minimizing systemic exposure (Uchida and Bruschi, 2023). Nevertheless, significant obstacles remain in scaling up 3D printing production for clinical application, requiring ongoing collaboration among materials scientists, engineers, and clinicians to streamline production processes and reduce costs.

### 6.5 Improving regulatory pathways

Despite promising progress in preclinical research, the clinical translation of natural polymer-based local anesthetic delivery systems still faces several hurdles. The biomedical industry must address technology transfer and process scaling, which necessitates substantial safety and efficacy data for clinical use. Partnerships among academic institutions, industry, and regulatory agencies are crucial for addressing these translational challenges (Li et al., 2023). The complex regulatory environment often prolongs approval timelines, so establishing clear regulatory pathways is also beneficial for accelerating the clinical translation of drug-loaded systems (Mohapatra et al., 2021). Legal aspects are also vital when promoting process scale-up, as they determine the success rate of industrial implementation from a regulatory perspective. For example, since May 26, 2022, hydrogels have been classified as Class III medical devices under new European regulations, requiring them to undergo a conformity assessment procedure before being marketed or put into service. This mandates the adoption of more detailed risk analysis and management specifications and imposes strict obligations on all market operators, thereby enhancing product transparency and traceability safeguards, and further promoting safety and reliability (Catoira et al., 2020).

## 7 Conclusions and future prospects

The research on natural polymer-based local anesthetic delivery systems has demonstrated significant progress, showcasing their immense potential to revolutionize perioperative pain management. Systems utilizing hyaluronic acid, chitosan, alginate, and cellulose have proven capable of providing sustained analgesia for periods ranging from hours to several days, effectively overcoming the major limitation of short duration associated with conventional local anesthetics. The excellent biocompatibility and biodegradability of these natural materials position them as safer alternatives to synthetic carriers, minimizing risks of long-term tissue accumulation and severe inflammatory responses.

However, the clinical translation of these promising systems faces several critical hurdles. Key challenges include the inherent limitations of individual polymers, such as the rapid enzymatic degradation of hyaluronic acid, the poor solubility of chitosan at physiological pH, and the weak mechanical strength of alginate hydrogels. Furthermore, achieving precise control over drug release kinetics to eliminate initial burst release and ensure predictable, long-term analgesia remains a significant obstacle. The scalability of manufacturing processes like 3D printing and microfluidics, while promising for personalization, must be addressed to meet Good Manufacturing Practice (GMP) standards for clinical use.

To fully realize the clinical potential of natural polymer-based delivery systems and overcome existing barriers, future research must adopt a cohesive and interdisciplinary strategy. The path forward should prioritize the development of intelligent composite materials through hybridization, such as creating chitosan-coated PLGA nanoparticles or alginate-gelatin blends, to synergistically enhance mechanical stability, control degradation rates, and improve drug-loading capacity. Building on this material foundation, there is a pressing need to engineer next-generation “smart” platforms that respond to specific physiological stimuli (e.g., pH, enzyme activity, ultrasound) for on-demand drug release; for instance, integrating microfluidics with ultrasound-responsive hydrogels could achieve a “three-in-one” system capable of precise release, long-lasting analgesia, and personalized dosing. Ultimately, accelerating clinical translation requires a concerted effort to bridge the gap between laboratory research and practical application, which entails establishing clear regulatory pathways for these complex products, conducting rigorous large-animal studies to validate long-term safety and efficacy, and developing scalable, cost-effective manufacturing technologies to ensure widespread availability.

In summary, while natural polymer-based delivery systems hold the key to a new era of safe and effective long-acting regional anesthesia, their success hinges on a targeted research strategy. By prioritizing intelligent material design, advanced fabrication technologies, and robust preclinical validation, we can accelerate the development of these innovative therapies to meet the evolving demands of clinical pain management.
